# Quantifying the impact of telemedicine and patient medical advice request messages on physicians' work-outside-work

**DOI:** 10.1038/s41746-024-01001-2

**Published:** 2024-02-14

**Authors:** Soumik Mandal, Batia M. Wiesenfeld, Devin M. Mann, Adam C. Szerencsy, Eduardo Iturrate, Oded Nov

**Affiliations:** 1 Dept of Population Health, New York University Grossman School of Medicine, New York, NY USA; 2 Technology Management & Innovation, New York University Tandon School of Engineering, New York, NY USA; 3 New York University Leonard N Stern School of Business, New York, NY USA; 4 MCIT Department of Health Informatics, NYU Langone Health, New York, USA

**Keywords:** Health services, Health policy

## Abstract

The COVID-19 pandemic has boosted digital health utilization, raising concerns about increased physicians’ after-hours clinical work ("work-outside-work”). The surge in patients’ digital messages and additional time spent on work-outside-work by telemedicine providers underscores the need to evaluate the connection between digital health utilization and physicians’ after-hours commitments. We examined the impact on physicians’ workload from two types of digital demands - patients’ messages requesting medical advice (*PMAR*s) sent to physicians’ inbox (inbasket), and telemedicine. Our study included 1716 ambulatory-care physicians in New York City regularly practicing between November 2022 and March 2023. Regression analyses assessed primary and interaction effects of (*PMAR*s) and telemedicine on work-outside-work. The study revealed a significant effect of *PMAR*s on physicians’ work-outside-work and that this relationship is moderated by physicians’ specialties. Non-primary care physicians or specialists experienced a more pronounced effect than their primary care peers. Analysis of their telemedicine load revealed that primary care physicians received fewer *PMAR*s and spent less time in work-outside-work with more telemedicine. Specialists faced increased *PMAR*s and did more work-outside-work as telemedicine visits increased which could be due to the difference in patient panels. Reducing *PMAR* volumes and efficient inbasket management strategies needed to reduce physicians’ work-outside-work. Policymakers need to be cognizant of potential disruptions in physicians carefully balanced workload caused by the digital health services.

## Introduction

Physician burnout and reduced work satisfaction are linked to increased electronic health records (EHRs) usage^1–3^, and specifically within the “inbasket,"^4,5^ where physicians exchange messages with patients and other healthcare providers. The inbasket, serving as the EHR’s communication hub, is a common functionality provided by EHR vendors. As physicians’ clinical workload increases, so does the amount of inbasket work, creating a burden that is challenging to manage during clinical hours^6^. To respond, physicians invest additional hours outside their official working hours, known as “work-outside-work"^7,8^ which exacerbates burnout. The inbasket work has been characterized as “Sisyphean"- an involuntary, ubiquitous, after-hours second job^4^, that can create a potentially 24/7 work environment for physicians which impacts their physical and mental wellness^9^. The increasing workload in inbasket may force the physicians to reduce clinical work and the time allocated to patients’ visits^10,11^, or leave medicine altogether creating further challenges for health services delivery that is already plagued by shortages of physicians^12,13^.

Physician time is a key resource in healthcare service delivery^11^. Already a challenge before the pandemic, physicians’ time in inbasket grew due to COVID-19-related disruptions^4^ that continue to transform how healthcare services are delivered^14^ and how patients access their routine care^15^. Patients have increasingly embraced digital health services such as telemedicine, patient portals and messaging applications that provide with greater access to their physicians^16–18^. A surge in telemedicine utilization, initially seen as a potential replacement for in-person visits, has evolved into a co-existing model as per more recent trends^15,19^, that may further increase physicians’ workload. Moreover, prior research suggests that physicians servicing higher percentage of appointments through telemedicine engage in increased levels of work-outside-work. This further suggests telemedicine, as currently delivered, is not as efficient, and likely to increase physicians’ work burden^8^. How increased telemedicine use leads physicians to spend more time in inbasket is unknown and requires in-depth investigation of their inbasket action logs.

On the other hand, patient portal messages are a new source of physicians’ asynchronous, electronic, non-visit care (NVC)^20^. Since the pandemic, patients have significantly increased their digital communication with their physicians^16^, leading the latter to spend more time in their inbasket^21^. To support physicians grappling with overloaded inbaskets, healthcare systems have deployed various inbasket management strategies such as, eliminating or suppressing messages that are of low clinical value, delegating, or reroute messages to other team members (e.g., advanced practice providers or APPs) for better inbasket coverage, adopting billing policies that would discourage patients from sending avoidable messages^22^, and developing smart functionalities (e.g., Macros) that enable physicians to “complete” inbasket messages more efficiently^16,23^ – each with a varying degree of effect^24^. While patients send messages to physicians’ inbasket for various reasons, such as prescription refills, appointment requests, and prior communication clarifications, the messages that are to “Get Medical Advice" (patient’s medical advice request, *PMAR*) often necessitate direct physicians’ review, making existing inbasket management strategies, such as delegating, and eliminating less effective. Despite the prior evidence on increasing *PMAR* messages in inbasket since pandemic^25^, existing research has only explored the broad impact of all inbasket messages on physicians’ workloads and burnout^5,9^. To understand the specific effects of increased *PMAR* messages on physicians’ time, further research is needed. Moreover, existing research on the impact of inbox messages has predominantly concentrated on primary-care physicians^9,26,27^. The influence of these messages on non-primary care physicians remains underexplored.

To address the above concerns, this study aims to investigate how the combined increase in *PMAR* messages and the expansion of telemedicine services, driven by COVID-19 disruptions, has impacted the amount of time physicians spend managing their inbasket. We examine the time spent in inbasket by ambulatory care physicians in one large health system (New York University Langone Health, NYULH) in New York City, and assess three key factors: (1) number of *PMAR* messages they received, (2) their specialties (whether they are primary care or non-primary care role), and (3) their telemedicine adoption, and the impact these factors have on their work-outside-work.

The study utilized five key measures: clinical load (*cl*), primary care (*pc*), *PMAR* messages received (*PMARs*), work-outside-work (*WOW*) and telemedicine intensity (*ti*). All measures were derived from data collected by Epic Signal, which reports average values per reporting period. Further details are provided in the Methods section.

## Results

### Descriptive analysis, provider specialties, clinical load

The majority of physicians in our cohort were from internal medicine subspecialties (e.g., cardiology, pulmonology), followed by ambulatory surgery (including general surgery and surgical subspecialists) and general medicine practice (e.g., family medicine). See Table 1 for further details. Among internal medicine subspecialties 289 physicians (16.84%) were practicing in Internal Medicine or Family Medicine department, and were treated as primary care physicians in the rest of the analysis. In total 240 cases involving approximately 4.6% of all physicians, total number of inbasket messages received by them were available, but data specifically on *PMAR*s were missing, which were estimated using multiple imputation.

**Table 1:** Specialty of physicians included in the study (*N*=1716)

Clinical specialty	Values (%)
Internal medicine subspecialty	725 (42.25)
General practice	241 (14.04)
Surgery	211 (12.30)
Pediatrics	132 (7.69)
Neurology	109 (6.35)
Obstetrician & gynecologist	73 (4.25)
Psychiatry	47 (2.74)
Dermatology	43 (2.51)
Emergency medicine	36 (2.10)
Rehab	20 (1.17)
Pain Medicine	5 (0.29)
Other	74 (4.31)

**Fig. 1:**
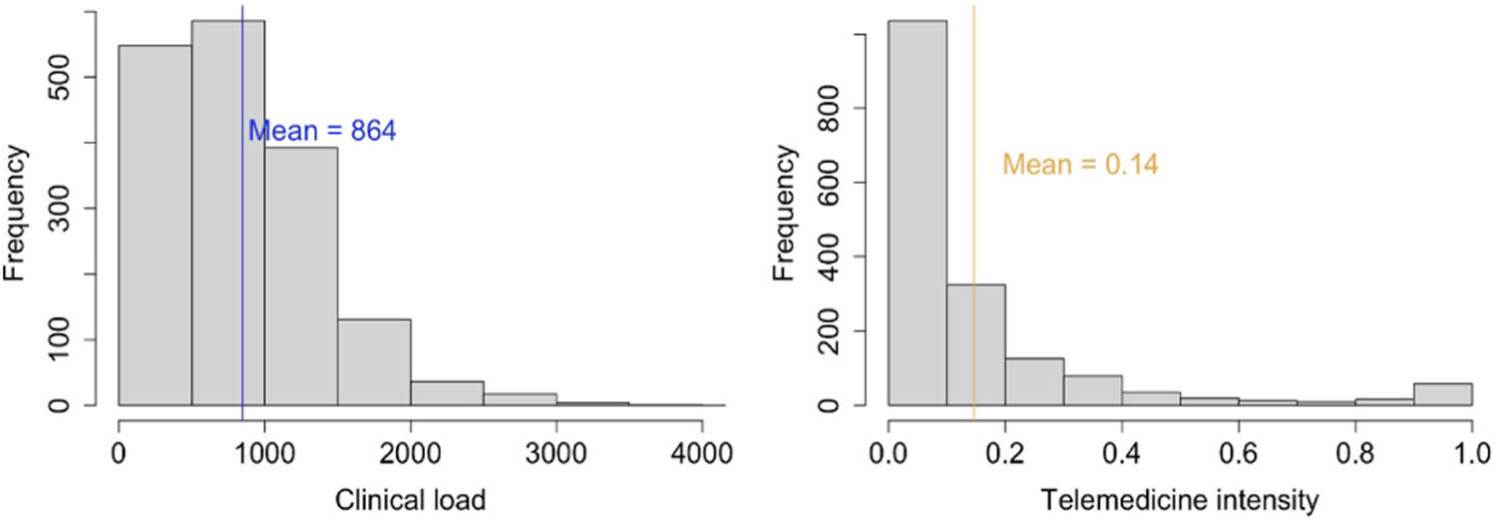
Histograms depicting distribution of physicians’ clinical load and telemedicine intensity.

Physicians on average completed 864 visits (min: 117, max: 4008, SD: 342.05) in the four months, with an average of more than 7 visits per day per physician. Majority (85.80%) of them were delivered in-person. The average proportion of total visits per physician that were telemedicine-based was 0.14 (SD: 0.21) (see Fig. 1). A multinominal logistic regression used to evaluate the change in *ti* over time (i.e., month of reporting) among physicians did not find any evidence of *ti* significantly changing during the study time period (F_1, 6716_ = 2.27, *p* value = 0.11).

**Fig. 2:**
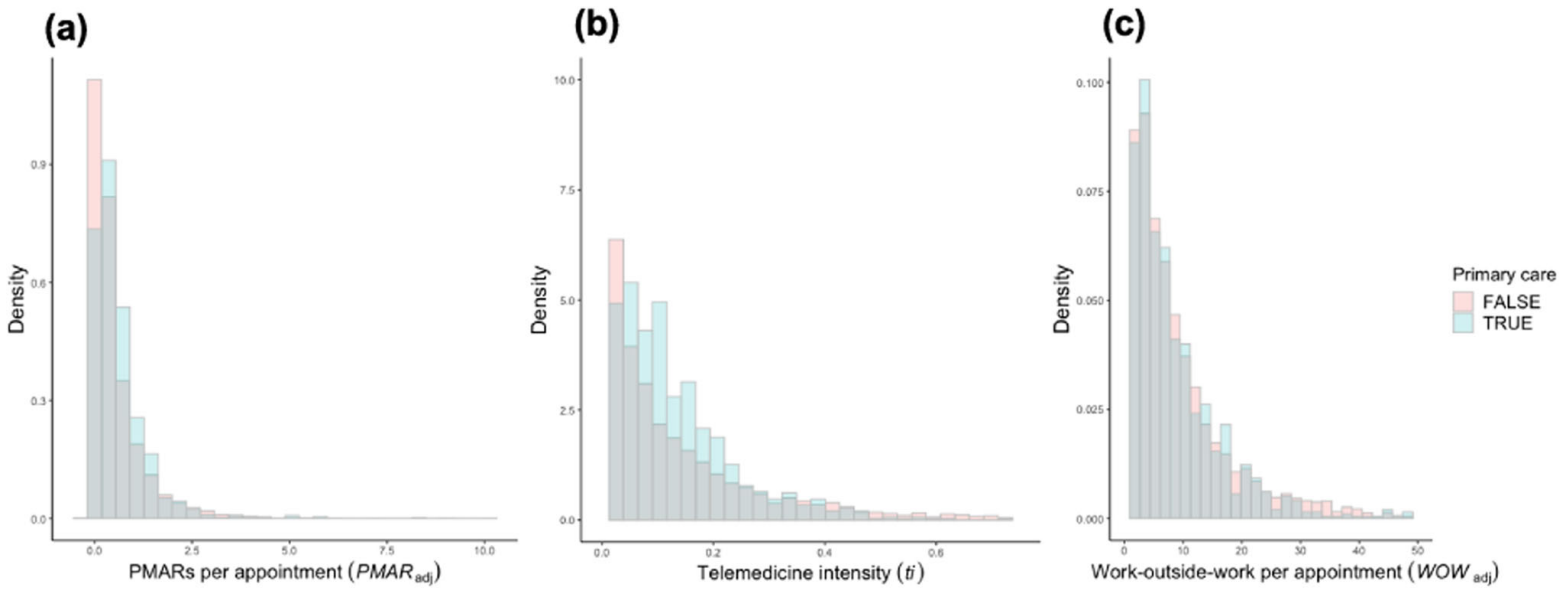
Distributions of key measures among primary care (n = 289) and non-primary care (n = 1427) physicians. **a** PMARs per appointment (*PMAR*_*ad*_); **b** telemedicine intensity (*ti*); **c** work-outside-work per appointment (*WOW*_*adj*_).

Our results showed physicians’ average *WOW*_*adj*_ was 9.48 minutes (min: 0.04 mins, max: 12.18 mins, median: 6.33 mins, SD: 10.46 mins). Additionally, the average *WOW*_*adj*_ was higher for non-primary care physicians (mean: 9.62mins, median: 6.37 mins) than their primary-care peers (mean: 8.80 mins, median: 6.02 mins). In contrast, primary care physicians, on average, received more *PMAR*s per appointment (mean: 0.61, median: 0.41), than their non-primary care physicians did (mean: 0.52, median: 0.26). Figure 2 shows the distributions of three continuous measures *PMAR*_*adj*_, *ti*, and *WOW*_*adj*_ among primary care and non-primary care physicians. One-way analysis of variance (ANOVA) found means of these two groups to be significantly different for all three measures.

### Association between key measures

Prior to investigating the effect of *PMAR*s and telemedicine intensity (*ti*) on physicians’ work-outside-work (*WOW*), we examined the association between these three measures using Pearson’s correlation coefficient. We discovered robust and positive correlations between physicians’ *PMAR* messages and their *WOW*. This association held true whether we considered unadjusted measures (coef. = 0.26, *p* value < 0.001, 95% CI [0.24, 0.29]) or measures adjusted (*PMAR*_*adj*_, *WOW*_*adj*_) for clinical load (coef. = 0.22, *p* value < 0.001, 95% CI [0.20, 0.24]). On the other hand, *ti* displayed a positive correlation with *PMAR*_*adj*_ (coef. = 0.13, *p* value < 0.001, 95% CI [0.10, 0.16]), but had a very small negative correlation with *WOW*_*adj*_ (coef. = −0.078, *p* value < 0.001, 95% CI [−0.13, −0.03]). Further investigation through dichotomizing *ti* based on median split revealed a more nuanced association between *ti* and *WOW*_*adj*_ - that for physicians with high *ti* had a negative association with the *WOW*_*adj*_ (coef. = −0.25, *p* value < 0.001, 95% CI [−0.33, −0.16]), whereas, for physicians with low telemedicine intensity, *ti* had a positive association with *WOW*_*adj*_ (coef. = 0.23, *p* value < 0.001, 95% CI [0.18, 0.29]). Overall, the findings suggest that *PMAR*s have a strong, positive relationship with physicians’ *WOW*, and that telemedicine may have a more nuanced effect on *WOW* depending on the physicians’ level of telemedicine load.

### Effect of *PMAR*s and telemedicine on work-outside-work

**Fig. 3:**
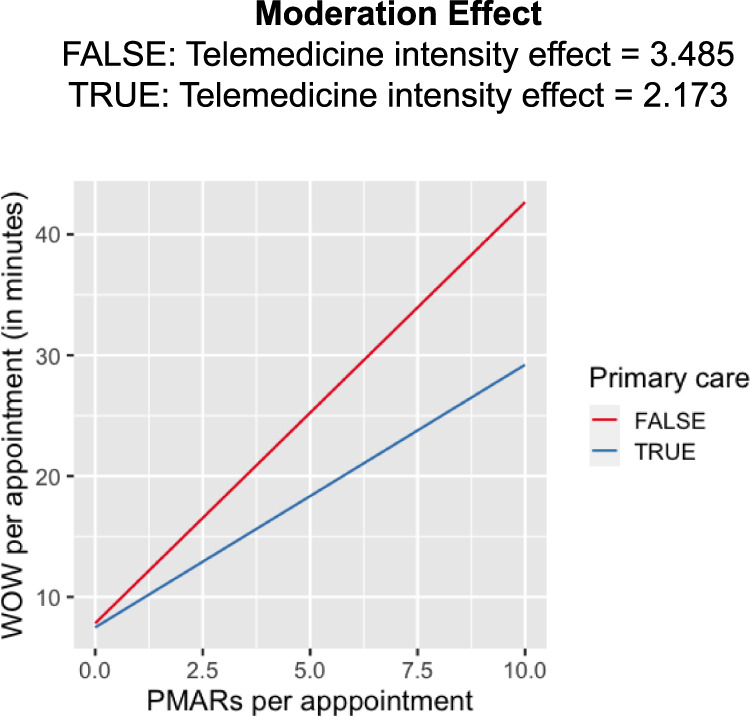
Predicted work-outside-work per appointment (*WOW*_*adj*_) based on primary-care and non-primary care physicians’ *PMAR*s per appointment (*PMAR*_*adj*_).

Our regression analysis found a statistically significant effect of *PMAR* volumes on the duration of time that physicians spend in *WOW*, irrespective of whether we considered the measures unadjusted (F_1,1716_ = 497, *p* value < 0.001) or adjusted for both measures at the per-appointment level (F_1,1716_ = 340, *p* value < 0.001). Additionally, our investigation also unveiled a significant disparity in the influence of *PMAR* per appointment (*PMAR*_*adj*_) between primary care and non-primary care physicians. As *PMAR*_*adj*_ increased, non-primary care physicians experienced a more pronounced rise in work-outside-work per appointment (*WOW*_*adj*_) compared to their primary care counterparts (see Fig. 3).

**Fig. 4:**
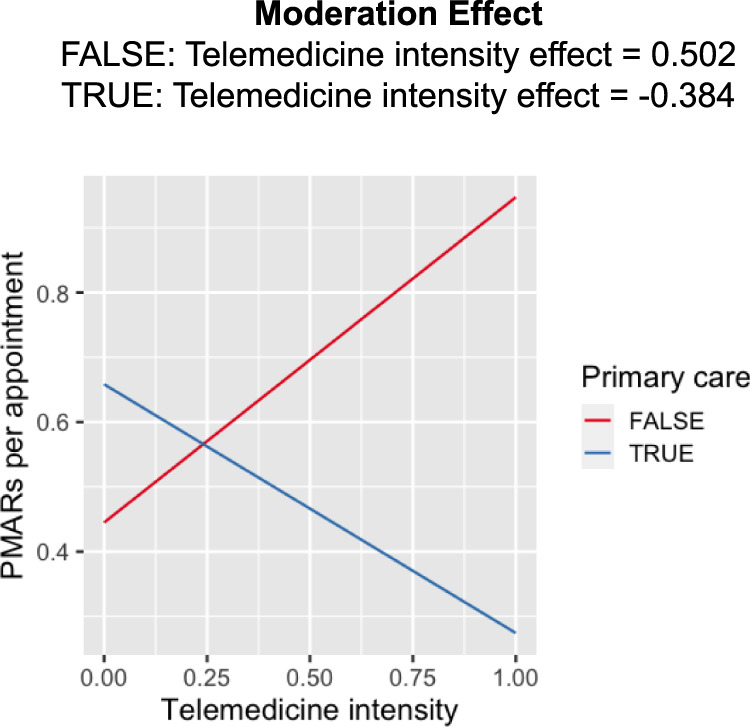
Predicted PMARs per appointment (*PMAR*_*adj*_) based on primary-care and non-primary care physicians’ telemedicine intensity (*ti*).

To evaluate the impact of telemedicine on *PMAR*s, we investigated the effect of telemedicine intensity (*ti*), primary care (*pc*) and their interaction on *PMAR* per appointment (*PMAR*_*adj*_). We found both the main effects of *ti* (coef: 0.50, Std. error: 0.04, *p* value < 0.001), and *pc* (coef: 0.21, std. error: 0.03, *p* value < 0.001) and their interaction (coef: −0.88, std. error: 0.16, *p* value < 0.001) to be significant. Our result showed that physicians’ specialties moderated the effect of telemedicine intensity on *PMAR* volumes. As the telemedicine intensity increased primary care physicians received less *PMAR*s per appointment, whereas physicians in non-primary care or specialties received more *PMAR*s with the increase in their *ti* (see Fig. 4).

**Fig. 5:**
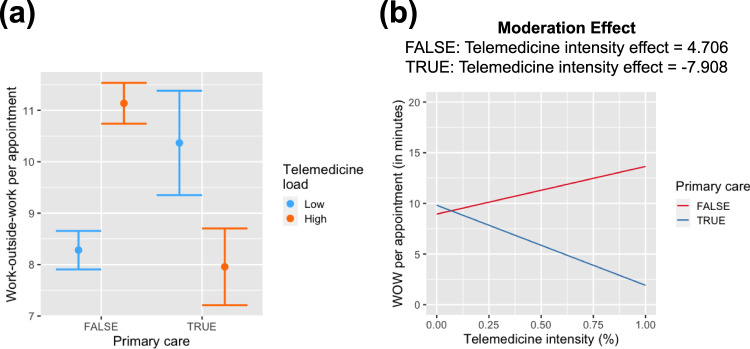
Effect of telemedicine service on work-outside-work per appointment (*WOW*_*adj*_) among primary care and non-primary care physicians. **a** Dichotomized telemedicine intensity (*ti*); **b** as a continuous measure.

To evaluate the effect on work-outside-work (Fig. 5), we conducted an analysis using a three-way interaction model, wherein the main effects of *PMAR*_*adj*_, *pc*, and *ti*, along with their interactions were evaluated for their potential impact on *WOW*_*adj*_. The results are summarized in Table 2. We did not find any evidence of the three-way-interaction significantly influencing *WOW*_*adj*_ (F_1,1716_ = 57.14, *p* value = 0.51). However, the two-way interaction between *ti* and *pc* was found to have a significant effect on *WOW*_*adj*_. Further investigating this two-way interaction between with physicians’ *ti* as both a continuous variable and dichotomized (high or low) based on median split (Fig. 5a), we consistently observed the interaction to have opposing effects based on physicians’ specialty. For primary-care physicians with a high telemedicine load, the *WOW*_*adj*_ attenuated, while for non-primary care physicians *WOW*_*adj*_ increased with the increase in physicians’ *ti* (see Fig. 5b).

**Table 2:** Three-way interaction result to estimate the effect of PMAR per appointment (*PMAR*_*adj*_), telemedicine intensity (*ti*), and physicians’ specialties (*pc*) on their WOW per appointment (*WOW*_*adj*_)

Variable	Estimate	Std. Error	*t* value	*p* value
Intercept	7.55	0.19	38.04	<0.001
*PMAR* per appointment (*PMAR*_*adj*_)	3.10	0.27	11.30	<0.001
Primary care (*pc*)	0.85	0.56	1.52	0.13
Telemedicine intensity (*ti*)	2.60	0.71	3.64	<0.001
*PMAR*_*adj*_ : *pc*	− 0.88	0.61	− 1.44	0.15
*PMAR*_*adj*_ : *ti*	0.91	0.77	1.18	0.24
*pc* : *ti*	− 9.08	2.79	− 3.26	0.001
*PMAR*_*adj*_ : *pc* : *ti*	− 2.51	3.78	− 0.66	− 0.51

## Discussion

The study aimed to evaluate the impact of two distinct digital health services on physicians’ workload: first, the level of patients’ digital medical advice request (*PMAR*) sent to physicians’ inbox (inbasket)), reflecting asynchronous clinical work, and second, physicians’ level of engagement in telemedicine services, intended to substitute some synchronous in-person work. Our findings reveal a significant influence of *PMAR*s on physicians’ work-related hours, evident in their engagement in after-hours clinical work (work-outside-work). Physicians with higher level of *PMAR*s spent more time on work-outside-work, a pattern that persisted after controlling for variations in physicians’ clinical load. The impact of *PMAR*s on work-outside-work varied across physicians’ departments or specialties. Notably, non-primary care physicians or specialists, with increased *PMAR*s, dedicated more time to work-outside-work compared to primary care physicians. Regarding telemedicine, our study shows telemedicine load has a more nuanced effect on physicians’ workload depending on their specialties and the proportion of their clinical time dedicated to telemedicine. In summary, while engagement in telemedicine has a significant (but waning than what previously reported^8,28^) effect on physicians’ effect on physicians’ work-outside-work, further analysis revealed contrasting effects on primary care and non-primary care physicians. Primary care physicians, with increased telemedicine visits, experience a decrease in *PMAR*s and work-outside-work. Conversely, non-primary care physicians receive more *PMAR*s, and subsequently, spend more time in work-outside-work as the proportion of telemedicine visits increased.

Health institutions have recognized physicians’ burnout concerns due to increasing inbasket workload, and are deploying strategies to address it^16,24^ e.g., filtering out low-clinical-value messages (elimination), delegation - rerouting messages to Advanced Practice Providers (APPs), and allocating dedicated resources to triage patient messages. Initial investigation suggests a positive effect of implemented strategies with a notable up to 75% reduction in certain message categories in physicians’ inbasket due to the elimination of unnecessary messages. Further research is needed to assess how these strategies have impacted non-primary care physicians and specialists with a particular focus on comparing their experiences with those of primary care physicians. Our overall observations show that non-primary care physicians or specialists dedicate more time to after-hours clinical work. The difference in patient panel among specialists could be a potential reason behind doing additional after-hours clinical work - PCPs typically manage ’fixed’ panel of mostly long-term patients, while specialists have a more dynamic mix of transient and long-term, necessitating additional time for EHR work. Future studies can explore the effect of panel size by comparing patient types between primary-care and non-primary care physicians.

Prior studies have suggested lack of familiarity with telemedicine^29^, difficulties due to switching frequently from one type of visit to another^30^, are some of the challenges physicians are facing with telemedicine. Whether these challenges affect specialties more severely than primary care that needs to be investigated. As our institution resumed in-person services post-pandemic waves, primary care practices shifted telemedicine use to less complex tasks that do not require patient’s physical presence, potentially explaining its attenuating effect on primary care physicians’ work-outside-work. Further investigation is needed into the utilization of telemedicine service in specialties to understand what factors contribute to non-primary care physicians providing more telemedicine services to receive more *PMAR*s. Future studies can investigate each of the potential factors independently in multiple cohort studies. Such investigations can inform the health organizations to create policies for optimizing the physicians’ schedule.

Recent work has advocated for a “digital minimalism” approach to addressing physician burden and burnout^31^. From this lens, healthcare organizations and policymakers need to be thoughtful of how their policies may inadvertently encourage *PMAR*s and the potential impact on physician workload. To reduce the burden of *PMAR*s on physicians, healthcare organizations should consider implementing policies and procedures that nudge patients to more carefully use the *PMAR* channel with their physicians, divert them to less burdensome tools, limit the number of non-urgent *PMAR*s or provide additional support and resources to help physicians manage their inboxes more efficiently. Potential optimizations might include enhanced digital self-service tools to answer *PMAR*s autonomously and AI/ML based models that automatically classify acuity from patient requests and triage the messages. Recent advancements in generative AI and large language models (LLMs) are being explored to draft responses that physicians could then edit^32^ for more efficient resolution of patient requests while maintaining quality and empathy in responses. Telemedicine must offer more than just marginal benefits for patients to justify physicians’ investment of time^28^. Future research should further investigate the relationships between subtypes of *PMAR*s and *WOW*/burden as well as the impact of the aforementioned optimization efforts.

The study has limitations, including the use of a dataset from a single EHR system, which limits the generalizability of the findings. Considering the widespread adoption of the same EHR vendor, Epic, globally, and the deployment of similar digital health technologies across numerous health system, comparable outcomes can be anticipated in a broad context. Limitations in our Epic Signal data set preclude the ability to review and analyze physician EHR activity with sufficient granularity beyond certain time periods; e.g., time periods more specific than a calendar month or physician activity log data at smaller than 15-minute increments. Our analysis is also constrained by the limited timeframe of data coverage, and the presence of seasonal variations in disease outbreaks, which can affect the results. Lastly, the study did not examine the effect of other factors that could affect physician workload, such as the complexity of patients’ conditions, their social determinants of health, physician characteristics (age, years in job, panel size, part-time vs full-time, writing style etc.) or the physicians’ work environment. Future in-depth investigation can control for these factors.

In conclusion, the study found that *PMAR*s and telemedicine have a significant impact on physicians’ workload, and telemedicine may have a more nuanced effect depending on the physicians’ level of telemedicine load. Our findings suggest that reducing *PMAR* volumes and improving the management of in-basket messages could help reduce physicians’ work-outside-work. Further research is needed to explore how *PMAR*s, and other digitally induced burdens affect physician workload to support the development of effective mitigation approaches.

## Methods

### Data source

Data was obtained as part of an EHR-based retrospective cohort study of 1716 ambulatory care physicians continuously practicing (defined as an average of at least one appointment per day in the reporting period) at any New York-based NYULH practice network for four months between November 27, 2022 - March 25, 2023. Non-physician practitioners (e.g., advanced-practice providers) and residents were expected to have different workflows and patient- facing responsibilities and thus, were not included in the study cohort. The data were obtained using Epic Signal, which provides comprehensive data on the time clinicians spent on different EHR interfaces based on user action logs. Additional EHR data on telemedicine visits that occurred during the specified time period and involved same set of physicians were extracted via Epic’s Clarity reporting database, aggregated to provider level and connected with the Signal data based on matching provider identifiers.

The study was deemed part of quality improvement and all data was collected as part of routine care. Ethics review and informed consent were not sought because the study met the criteria for exemption from such review according to NYULH institutional policy.

### Study measures

This section defines the key measures used in the analysis.

### Clinical load (*cl*)

Clinical load refers to the amount of clinical or patient care responsibilities that a healthcare provider is responsible for, and is typically measured panel size, percent of appointment slots filled^33^. Prior research has shown an association between clinical load and work-outside-work burden^7,34,35^. This study measured clinical load as the number of appointments or visits they completed during the reporting period. This was measured using the average number of appointments per clinical day as obtained from Signal, multiplied by number of clinical days (days with at least one appointment) in the reporting period.

### Primary care (*pc*)

A primary care practice serves as the patient’s entry point into the health care system. Prior research points to the magnifying workload of primary care physicians (PCP) as organizations transitioned to ambulatory care: the PCP is now expected to gain content on all patient encounters (whether initiated by PCP or not) and coordinate effectively^36^. This study used physicians’ department and specialty information available from Signal data to classify them as primary care and non-primary care. Physicians from internal medicine or family medicine were grouped as primary-care and the rest were treated as non-primary care physicians.

### PMAR messages received (*PMARs*)

This measure refers to the total volume of messages initiated by patients to “Get Medical Advice” that physicians receive exclusively in their inbasket during the reporting period. Messages that were sent to a shared inbasket (pool messages) and managed by multiple clinicians (APPs) were excluded from this count. Signal provides the number of such messages for a physician normalized by each day the physician logged into the system, and we calculated the total volume of *PMAR*s by multiplying the average daily number of such messages by the number of days the physician logged into the system during the reporting period.

### Work-outside-work (*WOW*)

The work-outside-work measure reflects after-hours clinical work of physicians based on the time they spend in the electronic health record (EHR) system outside of their scheduled patient hours^7,8^. To measure this, we combined the time spent in the system outside of scheduled hours on scheduled days (scheduled hours are determined from Epic Cadence scheduling data plus two 30-minute “buffer" periods added before the start of first appointment and after the end of last appointment) and time spent on unscheduled days (i.e., minutes spent in the system on days with no scheduled patients), and measured the work-outside-work in minutes. Although, Epic’s own metric *pajama time* (*PT*: the average number of minutes per day spent in charting activities on weekdays outside a standard 7 AM to 5:30 PM workday and any time on weekends) was considered as an alternate representation of after-hours clinical work, recent studies called into question *PT’*s appropriateness for this purpose^26^. Therefore, we did not use *PT* and instead relied on our own measure of work-outside-work.

### Telemedicine intensity (*ti*)

Telemedicine intensity reflects the proportion of clinical care the physician provides via telemedicine^8^. To compute telemedicine intensity, we categorized each patient appointment completed by a physician during the reporting period as either telemedicine or in-person using information queried from the EHR. Then, we aggregated all telemedicine visits for each physician and measured the proportion of telemedicine to total visits completed (numerator: number of telemedicine visits, denominator: total no of visits), resulting in a telemedicine intensity measure with a value that could range from 0 to 1.

### Analytical approach

In this section, we characterize different methods used in this study for understanding the relationship between key measures listed above, discussing the appropriateness of their respective assumptions. We first estimated clinical load (*cl*), and telemedicine intensity (*ti*) for all physicians in the EHR that met our inclusion criteria. Next, we adjusted the *WOW* and *PMAR* measures for variations in physicians’ *cl* and computed *WOW* per appointment (*WOW*_*adj*_) and *PMAR* per appointment (*PMAR*_*adj*_).

### Correlation analysis

We first analyzed the bivariate correlations to measure the association of *PMAR* and *WOW* both unadjusted and adjusted, and their association with telemedicine intensity (*ti*). We hypothesized that physicians’ *PMAR* would be positively associated with their *WOW*. The missing data points were addressed prior to this step. We assumed that for any physician with missing data on *PMAR* message volumes or *WOW* measures, data were missing at random (MAR)^37^, and therefore, adopted intent-to-treat analyses using multiple imputations to estimate the missing values^38^.

### Regression analysis

**Fig. 6:**
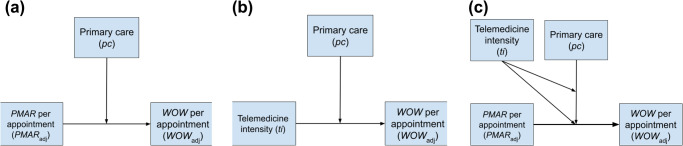
Conceptual models showing the relationships tested between key measures. **a** Interaction effect of the physicians’ specialties (pc) and *PMARs* per appointment (*PMAR*_*adj*_) on their work-outside-work per appointment (*WOW*_*adj*_) in minutes (H1); **b** interaction effect between telemedicine intensity (*ti*) and pc on *PMAR*_*adj*_ (H2); **c** three-way interaction model with pc as the primary moderator, and ti as the secondary moderator (H3).

To evaluate the effect of *PMAR* on *WOW*, we used multiple linear regression analysis in which *WOW* per appointment (*WOW*_*adj*_) was the dependent variable, and *PMAR* per appointment (*PMAR*_*adj*_) is the predictor. We further hypothesized (H1) that this effect varies based on the physician’s specialty. To address this, we introduced a binary variable (*pc*) to distinguish between primary-care and non-primary care physicians. Our analytical approach involved a hierarchical linear regression analysis, in which initially the main effects of *PMAR*_*adj*_ and *pc* were examined, followed by assessing their interaction (see Fig. 6a). Next, following the same hierarchical process, we measured the effect of interaction between telemedicine intensity (*ti*) and *pc* on *PMAR*_*adj*_. Our hypothesis (H2) was telemedicine generates more *PMAR*s and this relationship is moderated by physicians’ specialties (see Fig. 6b). Finally, we used a three-way interaction among *PMAR*_*adj*_, *pc*, and telemedicine intensity (*ti*) in predicting *WOW*_*adj*_. We hypothesized (H3) that the size or direction of the interaction effect between the *PMAR*_*adj*_ and *pc* on *WOW*_*adj*_ varies depending on the *ti* of the physician. In this model, *pc*c is a primary moderator, and *ti* is a secondary moderator (see Fig. 6c). All analyses were conducted in R statistical environment.

### Supplementary information

Supplementary document

## Data Availability

Access to the primary data used in this study requires a Data Use Agreement and IRB approval from the study institution (NYULH) and its EHR vendor (Epic). Subject to these requirements, aggregated data can be provided by the authors upon a reasonable request after de-identification. The secondary data on visit records used for analysis were sourced from the electronic health record (EHR) data of the New York University (NYU) Langone Health (NYULH) system, containing protected health information (PHI), and cannot be disclosed publicly.

## Code availability

All statistical analyses were performed in R (v4.2.1). The underlying code for this study performing the statistical analyses on the aggregated data sets is not publicly available due to the protection of the study data, but can be made available to qualified researchers on reasonable request from the corresponding author and requires a data use agreement.
